# A de novo next generation genomic sequence assembler based on string graph and MapReduce cloud computing framework

**DOI:** 10.1186/1471-2164-13-S7-S28

**Published:** 2012-12-07

**Authors:** Yu-Jung Chang, Chien-Chih Chen, Chuen-Liang Chen, Jan-Ming Ho

**Affiliations:** 1 Institute of Information Science, Academia Sinica, Taipei, Taiwan, ROC; 2 Department of Computer Science and Information Engineering, National Taiwan University, Taipei, Taiwan, ROC

## Abstract

**Background:**

State-of-the-art high-throughput sequencers, e.g., the Illumina HiSeq series, generate sequencing reads that are longer than 150 bp up to a total of 600 Gbp of data per run. The high-throughput sequencers generate lengthier reads with greater sequencing depth than those generated by previous technologies. Two major challenges exist in using the high-throughput technology for *de novo *assembly of genomes. First, the amount of physical memory may be insufficient to store the data structure of the assembly algorithm, even for high-end multicore processors. Moreover, the graph-theoretical model used to capture intersection relationships of the reads may contain structural defects that are not well managed by existing assembly algorithms.

**Results:**

We developed a distributed genome assembler based on string graphs and MapReduce framework, known as the CloudBrush. The assembler includes a novel edge-adjustment algorithm to detect structural defects by examining the neighboring reads of a specific read for sequencing errors and adjusting the edges of the string graph, if necessary. CloudBrush is evaluated against GAGE benchmarks to compare its assembly quality with the other assemblers. The results show that our assemblies have a moderate N50, a low misassembly rate of misjoins, and indels of > 5 bp. In addition, we have introduced two measures, known as precision and recall, to address the issues of faithfully aligned contigs to target genomes. Compared with the assembly tools used in the GAGE benchmarks, CloudBrush is shown to produce contigs with high precision and recall. We also verified the effectiveness of the edge-adjustment algorithm using simulated datasets and ran CloudBrush on a nematode dataset using a commercial cloud. CloudBrush assembler is available at https://github.com/ice91/CloudBrush.

## Background

With the rapid growth of DNA sequencing throughput delivered by next-generation sequencing technologies [1], there is a pressing need for *de novo *assemblers to efficiently handle massive sequencing data of genomes using scalable, on-demand, and inexpensive commodity cloud servers. *De novo *genome assembly is a fundamental step in analyzing a newly sequenced genome without a backbone sequence. *De novo *assembly software must deal with sequencing errors, repeat structures, and the computational complexity of processing large volumes of data [2]. The most recent assemblers use de Bruijn graphs [3-10] or string graphs [11-14] to model and manipulate the sequence reads. Using the de Bruijn graph model of sequence assembly requires breaking reads into short k-mers [3]. Typically, de Bruijn graph-based assemblers must recover the information lost from the breaking of reads, and attempt to resolve small repeats using read threading algorithms [14]. Using the string graph model of assembly can help avoid this issue. However, with the deeper coverage depth of read data, our preliminary studies show that the underlying string graph used to model the intersection of reads becomes much more complex than expected by previous assembly algorithms [15].

**Figure 1:**
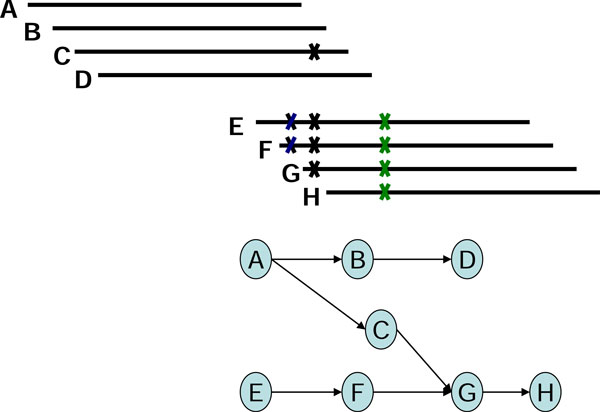
**The chimerical link structure C-G in a string graph**.

**Figure 2:**
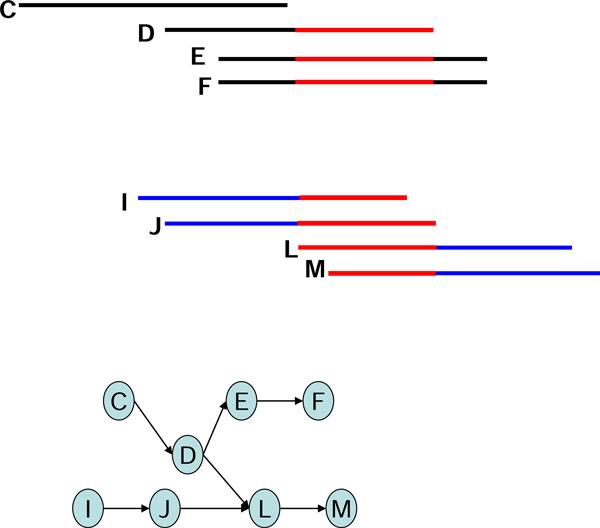
**The short-repeat branch D-L in a string graph**.

**Figure 3:**
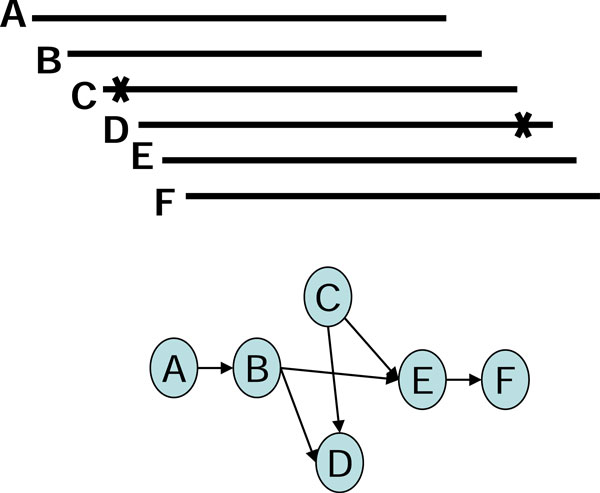
**The braid structure in a string graph**.

After building the assembly graphs, algorithms based on de Bruijn graphs or string graphs manipulate the graph-theoretic models by using several operations of graph simplification to repair erroneous reads and to remove redundancy in graphs, such as removing short dead-end tips and bubbles of similar paths [2]. Erroneous reads and repeats may also result in more compounds with branch structures that complicate the assembly, especially as the sequencing depths of reads become greater and error rates increase. One example of the challenges faced is the chimerical links of edges, also known as chimerical connections [4], formed by partial overlap of two unrelated contigs (Figure 1), where the partial overlaps are caused by sequencing errors. Other examples are ambiguous branching caused by short repeats and "braids" formed by shared branches (Figures 2, 3).

To unfold these complex branch patterns into correct linear paths in string graphs, we present an Edge Adjustment (EA) algorithm to remedy this problem. The algorithm utilizes the sequence information of all graph neighbors for each read and eliminates the edges connecting to reads containing rare bases. We also used simulated read datasets of *Escherichia coli *genomes of varying sequencing depths and error rates to verify the effectiveness of the EA algorithm. In addition, we integrated the EA algorithm into a distributed assembly program [15] based on string graphs and MapReduce cloud computing framework [16,17], known as CloudBrush. We evaluated the method against the GAGE benchmarks established by Salzberg et al [18] to compare assembly quality with other *de novo *assembly tools. Moreover, we introduced a pair of novel indices to measure the quality of sequence assembly, known as precision and recall, to indicate whether the output contigs are faithfully aligned (i.e., without inversions or rearrangements) with a contiguous region in the target genome, and whether the output contigs fully cover the entire target genome. It is noteworthy that these two indices are important for follow-up annotation and analysis of the target genome. Finally, we ran CloudBrush on a nematode dataset using a computing cloud [19] and analyzed its performance.

## Results

### Structural defects in string graphs

In using the graph-based assembly approach, sequencing error may generate complex structures in the graph. For example, sequencing errors at the end of reads may create tips in the graph, and sequencing errors within long reads may create bubbles in the graph. Tips and bubbles are well-defined problems with a solution making use of the topological features of the graph as described in [4] and [10]. Some errors, however, create more complex structures that cannot be readily identified from the topology of the graph. In this report, we refer to these structures are "structural defects." A well-known structural defect is the chimerical link problem. Figure 1 displays an example of chimerical links caused by sequencing error in string graph. In this instance, the chimerical link is caused by false overlap between node C and node G. In addition to sequencing errors, repeat regions also cause structural defects in a string graph; for example, the well-known "frayed rope" pattern [2]. Furthermore, repeats shorter than the read lengths may also complicate processing in string graphs; for example, if a short repeat exists in reads D, E, F, I, J, L, and M, where C, D, E, and F are reads from a specific region in the genome, while I, J, L, and M are reads from another region in the same genome (Figure 2). It is noteworthy that in the string graph, the edge between nodes D and L is denoted as a "branch structure" which may lead an assembly algorithm to report an erroneous contig. In addition to false overlaps, missing overlaps also introduce structural defects into the string graph; for example, the formation of a braid structure caused by sequencing errors appearing in continuous reads (Figure 3). In this instance, two missing overlaps forbid the adjacent reads from being merged together; node B lost an overlap link to node C, and node D lost an overlap link to node E (Figure 3). Similar to the chimerical link problem, it is challenging to use topological features of the graph to deal with braid structures.

### Edge Adjustment with the neighbors' contents

**Figure 4:**
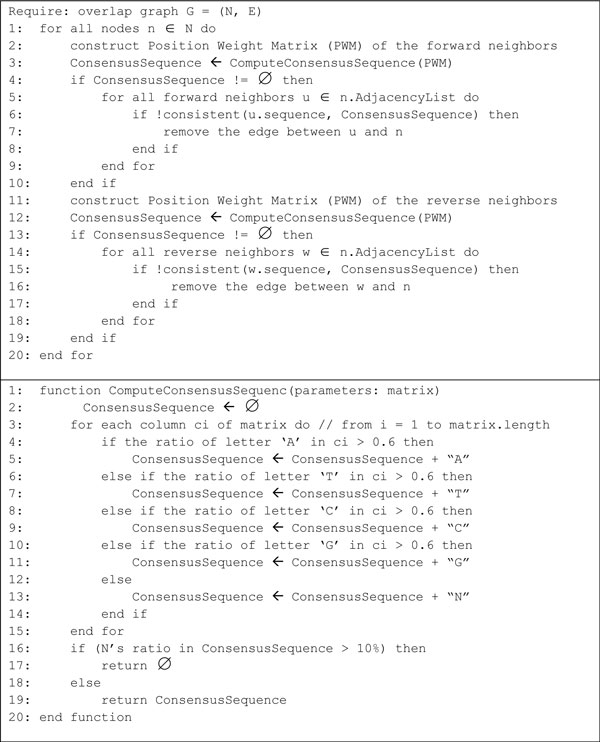
**The pseudo code of the EA algorithm in sequential version**.

**Figure 5:**
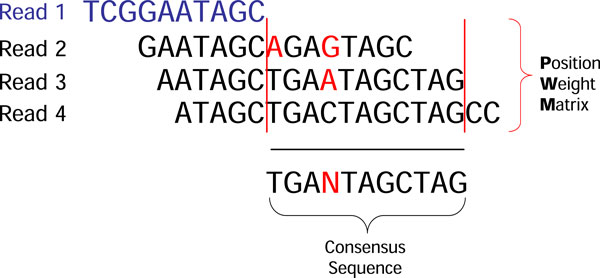
**The illustration of position weight matrix**.

We present the Edge Adjustment (EA) algorithm to fix structural defects in string graphs. For a node *n *in the string graph *G*, the EA algorithm adjusts edges of *n *by examining neighbors of *n *to decide whether each neighbor has sequencing errors or not. Figure 4 shows the pseudo code of the Edge Adjustment algorithm in sequential version. Note that we are dealing with NGS reads with the same length. Thus neighbors of *n *may be divided into two groups, i.e., forward neighbors and reverse neighbors. A forward neighbor of *n *overlaps with the suffix of *n*; while a reverse neighbor of *n *overlaps with the prefix of *n*. To construct node *n*'s Position Weight Matrix (PWM) of its neighbors in one of the two directions, we first align the reads of the neighbors to *n*. Then, we use the subsequences of each read ranging from the end of node *n *to the end of the second-last neighbor to define PWM of *n*. A consensus sequence of neighbors can be obtained by computing the PWM of the neighbors. PWM has four rows corresponding to A, T, C and G respectively. An element of PWM in column *i *is the number of occurrences of *β *at position *i*, where *β*∈{A, T, C, G}. We may then define the consensus sequence of these subsequences as follows:

(1) $$Consensus_{i}=\left\{\begin{array}{c} \beta_{i}|\beta_{i}/S_{i}>0.6\\ \prime N\prime |\forall \beta_{i}/S_{i}\leq 0.6\;\; \end{array}\right\}$$

where *i *represents the position in the consensus sequence corresponding to the column position in PWM; *β*∈ {A, T, C, G}; *β_i _*is the number of occurrences of *β *at position *i*; and *S_i _*is the sum of occurrence of letters at position *i*. We use the letter 'N' at position *i *of the consensus sequence, if for every letter in {A, T, C, G}, we have *β_i _/S_i _*≤ 0.6. Note that if the percentage of 'N' in the consensus sequence is greater than 10%, then this consensus sequence is rejected by the EA algorithm and all neighbors in the specific direction are retained. Otherwise, the consensus sequence is used to detect sequencing errors in each neighbors *n' *of *n *by comparing the subsequence of *n' *with the consensus sequence. The edge *(n, n') *is removed if the subsequence of *n' *is found inconsistent with the consensus sequence. In our experiment, the subsequence of *n' *is said to be *consistent *with the consensus sequence if every character of the subsequence is equal to the character, except character 'N', on the consensus sequence at the same position. Note that, for each node of the string graph, the EA algorithm generates a consensus sequence for each direction to perform the consistency check and to remove edges which are inconsistent with the consensus sequence. In an illustration of an EA algorithm, read 1 has three neighboring reads: 2, 3, and 4 (Figure 5). The range of the PWM exists from the end of read 1 to the end of read 3. Since read 2 has a character 'A' which is different from the first character 'T' of the consensus sequence (Figure 5), the edge between read1 and read2 will be removed. Next, we use the following examples to illustrate the reduction of structural defects in a string graph by using the EA algorithm.

**Figure 6:**
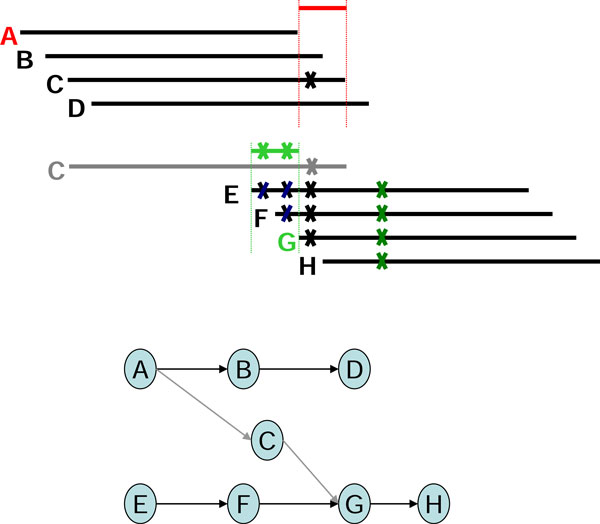
**The example of the chimerical link structure was solved by using Edge Adjustment**.

**Figure 7:**
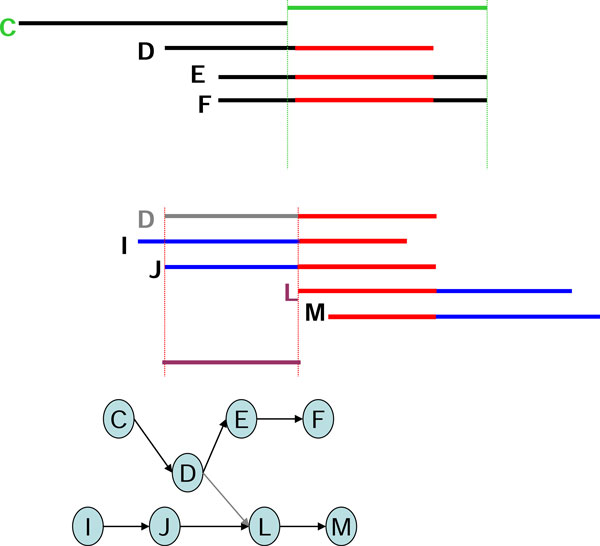
**The example of the branch structure was solved by using Edge Adjustment**.

**Figure 8:**
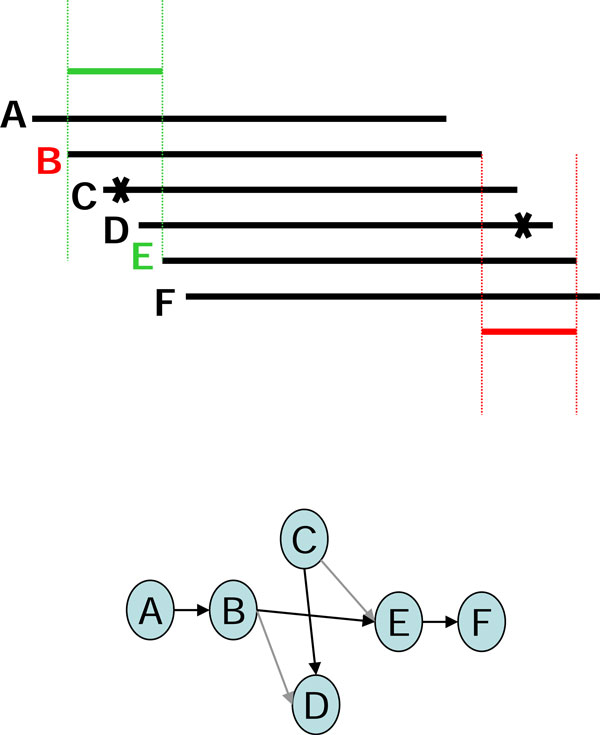
**The example of braid structure was solved by using Edge Adjustment**.

One example each of a chimerical link problem, a branch structure problem, and a braid problem that were solved with the EA algorithm are displayed (Figures 6, 7, 8). To solve the chimerical link problem, the EA algorithm generates a consensus sequence for read A (shown in red) from the neighboring reads B, C, and D (Figure 6). Since read C has one character that is different from the consensus sequence, the overlap link between reads A and C will be removed. By contrast, the EA algorithm generates a consensus sequence for read G (shown in green) from the neighboring reads C, E, and F (Figure 6). Thus, the overlap link between reads C and G will be removed in a similar manner.

To solve the branch structure problem, the EA algorithm generates a consensus sequence for read L from the neighboring reads D, I, and J (Figure 7). Therefore, read D differs from the consensus sequence, which is primarily represented by reads I and J. The overlap link between reads D and L is removed.

To solve the braid structure problem, in which instance the errors in reads C and D complicate the graph structure, the EA algorithm removes the overlap links between reads C and E and between reads B and D (Figure 8). Thus, reads C and D are isolated from the main graph, and no braid structure exists.

### Analysis of edge adjustment

We prepared simulated datasets generated from the *E. coli *genome to evaluate effectiveness of the EA algorithm. In other words, the position of each read on the target genome, and thus positions of sequencing errors on the read are also present in the dataset. We subsequently construct the overlap graph of the dataset by creating a node to present each read, and an edge between each pair of reads if they have a sequence overlap with size no smaller than an integer *k*. Two attributes are associated with each edge of the overlap graph from the simulated data. In the first attribute, if the positions of the two reads overlap with each other on the genome, then the overlapping region is designated as a *true *edge; otherwise, it is designated as a *false *edge. The second attribute is used to denote whether any sequencing error exists on the two reads of the edge. Therefore, we can now classify edges of the overlap graph into four classes according to these two attributes. Class I denotes the subset of *true *edges without sequencing errors; class II denotes the subset of true edges with sequencing errors; class III denotes the subset of *false *edges with sequencing errors; and class IV denotes the subset of *false *edges without sequencing errors. It is noteworthy that class I edges are most desired to improve the quality of data for subsequent stages of sequence assembly. By contrast, class III edges are chimerical edges; class II edges contain sequencing errors; and class IV edges contain reads that intersect repeats. Edges of classes II, III, and IV may introduce errors or structural defects into the later stages of sequence assembly. Therefore, it is the design goal of the EA algorithm to minimize the number of class II, III, and IV edges and to maximize the number of class I edges.

To test the effectiveness of the EA algorithm, we generated four sets of simulated data. In the first and second sets, 36-bp reads were generated at a constant coverage depth of 100×, and single base errors were inserted at rates of 0.5% and 1%, respectively. In the third and fourth sets, 150-bp reads were generated at a constant coverage depth of 200×, and single base errors were inserted at rates of 0.5% and 1%, respectively. Table 1 shows the number of edges of the overlap graphs before and after performing the EA algorithm. We observed that most of the edges removed by EA algorithm were class II edges (i.e., possessing sequencing errors). We also observed that the EA algorithm was quite effective in removing class III (chimerical) edges for the two 150-bp datasets, and satisfactory in removing the class III edges for the two 36-bp datasets. By contrast, only about 20% of the class IV edges (i.e., those containing reads that intersect repeats) are removed by the EA algorithm.

**Table 1:** The edge analysis of overlap graph before and after Edge Adjustment

Simulated *E. coli *Dataset	Edge Type	# of edges before Edge Adjustment	# of edges after Edge Adjustment	
100 × 36 bp 0.5% error dataset	Class I	92829732	92754696	[99.92%]
	Class II	14519426	322510	[2.22%]
	Class III	252762	118542	[46.90%]
	Class IV	377856	294110	[77.84%]

100 × 36 bp 1% error dataset	Class I	76439532	76364264	[99.90%]
	Class II	24836446	749900	[3.02%]
	Class III	358432	76162	[21.25%]
	Class IV	132412	92834	[70.11%]

200 × 150 bp 0.5% error dataset	Class I	115230002	115163888	[99.94%]
	Class II	74214420	438274	[0.59%]
	Class III	1347100	51988	[3.86%]
	Class IV	403836	322746	[79.92%]

200 × 150 bp 1% error dataset	Class I	32604042	32580388	[99.93%]
	Class II	53758272	554020	[1.03%]
	Class III	1422472	57494	[4.04%]
	Class IV	256952	225124	[87.61%]

We define a braid index to provide an approximate measure of the number of braid structures in a set *S *of reads. To acquire the braid index, we first constructed the overlap graph *G^o^(S) *of *S*. We next constructed a simplified string graph *G^s^(S) *of S which is obtained from *G^o^(S) *by removing contained reads, transitive edges, and concatenating, "one-in one-out" nodes. For each node *v *of *G^o^(S)*, we next examined its neighborhood for a pair of vertices, *u_1 _*and *u_2_*, and an additional vertex *v'*, such that the following properties exist: (1) (*u_1_, u_2_*) is not an edge of *G^o^(S)*; (2) *u_1 _*and *u_2 _*form a consensus when both are aligned to *v*; (3) *(v, v') *is not an edge of *G^o^(S); *and (4) *v *and *v' *form a consensus when aligned to *u_1 _*and *u_2_*. The braid index is then defined as the number of tuples *(v, v', u_1_, u_2_) *satisfying the aforementioned four properties. Table 2 shows the braid indices of the simplified string graphs of the four data sets with and without the performance of the EA algorithm. We observed that a dataset with a larger sequencing error has a larger braid index and may therefore possess more complicated braid structures. By contrast, the EA algorithm has also been shown to be effective in removing braid structures.

**Table 2:** The analysis of simplified string graph with and without Edge Adjustment

Simulated Data	Graph feature	without Edge Adjustment	with Edge Adjustment
100 × 36 bp 0.5% error dataset	# of node	2502312	1572470
	# of edge	2220162	26079
	braid index	342736	750

100 × 36 bp 1% error dataset	# of node	4418943	2964253
	# of edge	4051264	46649
	braid index	873835	802

200 × 150 bp 0.5% error dataset	# of node	3839687	2680727
	# of edge	6618017	7739
	braid index	1750824	242

200 × 150 bp 1% error dataset	# of node	5085964	4245557
	# of edge	8501560	16767
	braid index	2350695	413

### Evaluation of assembly accuracy

Hypothetically, a perfect assembly result produces nothing but subsquences of the reference sequences. In particular, rearrangements do not exist in any contigs. To distinguish superior assembly results from those containing collapsed repetitive regions or rearrangements, we designed a strict measurement scheme known as precision and recall. The precision and recall focus on the quality of the contigs. A contig must be aligned along its whole length with a base similarity of at least 95% in order to be considered valid. The union of all the valid contig areas in the references was treated as a true positive, and the recall was defined using the following formula:

(2) $$\text{Recall}=\frac{\text{number of true positive bases in reference }}{\text{total length of reference sequence}}$$

Similarly, the union of all the valid contigs areas on the side of contigs was treated as a true positive in contigs, and the precision was defined using the following formula:

(3) $$\text{Precision}=\frac{\text{number of true positive bases in contigs}}{\text{total length of contigs}}$$

Importantly, we only evaluate contigs whose length ≥ 200 bp.

We used three real and two simulated datasets to test CloudBrush and the other assemblers. The first real dataset was a set of short read data from an *E. coli *library (NCBI Short Read Archive, accession no. SRX000429) consisting of 20.8 M 36-bp reads. The second real dataset was released by Illumina, which included 12 M paired-end 150-bp reads. This dataset contains sequences from a well-characterized *E. coli *strain K-12 MG1655 library sequenced on an Illumina MiSeq platform. For the two real datasets, we select the first half of reads to evaluate assemblers, and their coverage depth was 81× and 197×, respectively. We used D1 and D2 to denote the 36-bp and 150-bp datasets, respectively. Furthermore, we downloaded *Caenorhabditis elegans *sequence reads (strain N2) from the NCBI SRA (accession no. SRX026594) as the D3 dataset, consisting of 33.8 M read pairs sequenced using the Illumina Genome Analyzer II and a constant coverage depth of 67×. The two simulated datasets were generated at random from the *E. coli *K-12 genome using 36-bp reads with 100× coverage depth and 1% mismatch errors, and with 100-bp reads with 200× coverage depth and 1% mismatch errors.

We performed assemblies on these datasets using Edena [12], Velvet [4], Contrail [10] and CloudBrush assemblers. Edena is the first string graph-based assembler for data of short reads. Velvet is one of the first de Bruijn graph-based assemblers for short reads that is often used as a standard tool for assembling small- to medium-sized genomes. Contrail is the first de Bruijn graph-based assembler using the MapReduce framework. Each assembler is required to set the parameter *k*, i.e., the minimum length of overlap for two contigs to form a longer contig. Considering the relationship between parameter *k *and coverage depth [20], we used *k *= 21 on dataset D1 and 100× simulated data, *k *= 75 on dataset D2 and 200× simulated data, and k = 51 on dataset D3. Importantly, we did not use pair-end information in this experiment.

**Figure 9:**
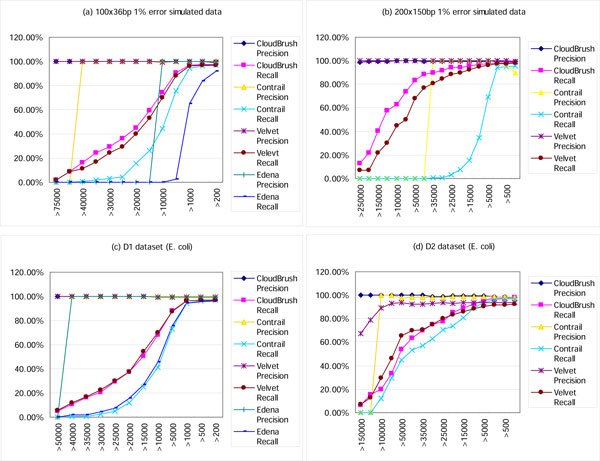
**The variation of precision and recall with different lower bounds of length on simulated data and datasets D1 and D2**.

Figure 9 shows the precision and recall of contigs with different length thresholds on the two simulated datasets of *E. coli *genome with a 1% error rate and datasets D1 and D2. We observed that CloudBrush outperforms the others for the two simulated datasets; the other assemblers generated more mis-assembly contigs when reads become longer from 36 bp to 150 bp (Figures 9a and 9b). For datasets D1 and D2, CloudBrush have similar performance of precision and recall leading the other assemblers (Figures 9c and 9d). Since longer reads and a larger error rate may generate more complex structure defects. CloudBrush may have a greater ability to handle complicated graph structures by using the EA algorithm.

We considered a number of different evaluation criteria, which are summarized in Tables 3 and 4. It is noteworthy that CloudBrush and Contrail ran on a cluster with 150 nodes each having 2 core CPU and 4 GB of RAM; while Edena and Velvet ran on a single machine which has 16 core CPU and 128 GB of RAM. Besides, Edena failed to work on datasets D2 and D3 in longer read data; therefore, no results were generated. Furthermore, we computed precision and recall by parsing the result of MegaBLAST [21].

**Table 3:** Evaluation of assemblies of the simulated dataset (100×, 36 bp, 1% error) and dataset D1 with CloudBrush, Contrail, Velvet, and Edena

Dataset	Assembler	# of contigs^1^	N50	Largest contig size	Precision	Recall	# of valid contigs^1^	# of invalid contigs^1^	Runtime (sec)
100 × 36 bp 1% error	CloudBrush	447	17907	95387	99.79%	97.51%	420	27	6218
	Contrail	906	8982	40066	99.72%	96.76%	858	48	5499
	Velvet	507	15632	100501	99.68%	96.95%	498	9	590
	Edena	4012	1436	11264	98.84%	91.85%	3868	144	2524

D1 dataset	CloudBrush	521	15149	66832	99.26%	97.10%	481	40	5555
	Contrail	930	8605	40066	99.73%	96.81%	886	44	4789
	Velvet	505	15862	73042	99.62%	96.90%	494	11	452
	Edena	889	9045	44942	99.18%	96.34%	823	66	1401

^1 ^Contigs with lengths > 200 bp are counted.

**Table 4:** Evaluation of assemblies of the simulated dataset (200 × 150 bp, 1% error) and dataset D2 and D3 with CloudBrush, Contrail, and Velvet

Dataset	Assembler	# of contigs^1^	N50	Largest contig size	Prec -ision	Recall	# of valid contigs^1^	# of invalid contigs^1^	Runtime (sec)
200 × 150 bp 1% error	CloudBrush	229	112531	327245	99.20%	96.00%	152	77	10616
	Contrail	2540	7554	36335	90.12%	95.92%	957	1583	15823
	Velvet	209	78642	327101	99.63%	98.10%	168	41	1317

D2 dataset	CloudBrush	361	52961	156592	98.10%	98.15%	230	131	8622
	Contrail	300	43609	124089	98.47%	96.98%	250	50	7200
	Velvet	189	71764	174184	93.60%	92.20%	164	25	927

D3 dataset	CloudBrush	37064	8880	114585	93.65%	92.41%	24603	10387	48603
	Contrail	31870	8274	105244	96.99%	90.89%	25236	6116	44619
	Velvet	23565	10847	106863	95.55%	89.01%	20187	2838	13963

^1 ^Contigs with lengths > 200 bp are counted.

### Comparison with other tools using GAGE benchmarks

To provide a comprehensive comparison, we used the benchmarks of GAGE [18] to evaluate CloudBrush and compared it with eight assemblers that were evaluated in GAGE benchmarks. Since GAGE provides the assembly results for each assembler, we used the precision and recall to evaluate each assembler to complement the evaluation of GAGE. Tables 5 and 6 summarize the validation results for the two genomes *Staphylococcus aureus *and *Rhodobacter sphaeroides*.

**Table 5:** Evaluation of *S aureus *(genome size 2,872,915 bp)

Assembler	Num	N50 (kb)	N50 corr. (kb)	Indel > 5 bp	Misjoins	Precision	Recall	# of valid contigs (> 200 bp)	# of invalid contigs (> 200 bp)
ABySS	302	29.2	24.8	9	5	75.06%	94.31%	219	83
ALLPATHS-LG	60	96.7	66.2	12	4	93.35%	92.28%	55	5
Bambus2	109	50.2	16.7	164	13	63.20%	61.69%	90	19
MSR-CA	94	59.2	48.2	10	12	90.14%	88.96%	79	15
SGA	1252	4	4	2	4	97.95%	95.61%	1134	118
SOAPdenovo	107	288.2	62.7	31	17	60.22%	60.35%	59	48
Velvet	162	48.4	41.5	14	14	82.66%	81.08%	136	26
CloudBrush	527	9.7	9.5	2	10	96.72%	96.00%	447	80

**Table 6:** Evaluation of *R. sphaeroides *(genome size 4,603,060 bp)

Assembler	Num	N50 (kb)	N50 corr. (kb)	Indel > 5 bp	Misjoins	Precision	Recall	# of valid contig (> 200 bp)	# of invalid contig (> 200 bp)
ABySS	1915	5.9	4.2	34	21	79.78%	86.13%	1744	171
ALLPATHS-LG	204	42.5	34.4	37	6	81.49%	81.22%	183	21
Bambus2	177	93.2	12.8	363	5	48.65%	46.21%	129	48
CABOG	322	20.2	17.9	24	10	92.55%	85.21%	310	12
MSR-CA	395	22.1	19.1	32	10	93.35%	90.55%	363	32
SGA	3066	4.5	2.9	4	4	97.23%	94.56%	2758	308
SOAPdenovo	204	131.7	14.3	406	8	70.86%	70.75%	134	70
Velvet	583	15.7	14.5	27	8	94.41%	92.37%	545	38
CloudBrush	661	12.8	12.7	10	2	96.21%	95.85%	567	94

As described in [18], a more aggressive assembler is prone to generate more segmental indels as it strives to maximize the length of its contigs, while a conservative assembler minimizes errors at the expense of contig size. We observed that the SGA assemblies have the fewest errors of misjoins and indels of > 5 bp, but have the shortest N50 (Tables 5 and 6). CloudBrush generated the second fewest number of errors, but led to a longer N50, which identified CloudBrush as a conservative assembler that could still assemble longer contigs.

A caveat on the use of the assembly precision and recall for contigs is required. When misjoined errors occur in a very long contig, the whole contig will be invalidated, and the precision and recall will obviously decrease in proportion to the contig length. By contrast, when misjoined errors occur in a shorter contig, the precision and recall may only decrease slightly. We observed that SGA and CloudBrush produced the highest precisions and recalls (Tables 5 and 6), indicating that the contigs generated will have very few artificial breakpoints generated by assemblers; moreover, it will reduce the noisy interrupts in the subsequent genome annotation and comparative genomic analysis. It is noteworthy that some assemblers e.g., Bambus2 [22] and SOAPdenovo [8], have lower precision and recall due to the fact that their misjoined errors and longer indels occur in longer contigs.

### Run time analysis

**Figure 10:**
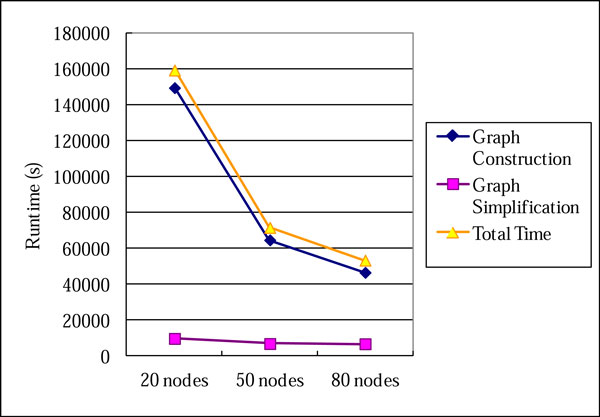
**Runtime analysis of Dataset D3 (*C. elegans*) by CloudBrush**.

To evaluate the performance of our approach, we performed CloudBrush analysis on three different sizes of Hadoop clusters using machines leased from the hicloud [19]. The three clusters consisted of 20, 50, and 80 nodes, respectively. Each node had 2 virtual CPUs (each one is equivalent to 1 GHz2007 Xeon processor) and 4 GB of RAM. We used the dataset D3 of *C. elegans *as the benchmark to analyze the runtime of CloudBrush. CloudBrush is counted separately in two phases: Graph Construction and Graph Simplification. We observed that the Graph Construction is the primary bottleneck of CloudBrush with 20, 50, or 80 nodes (Figure 10). However, with an increase in the number of nodes, the computation time of Graph Construction decreases substantially, while the runtime of Graph Simplification decreases only slightly. Using 20 nodes as a baseline, when the number of nodes is increased 2.5-fold, the construction time is decreased 2.3-fold and the simplification time is decreased by 1.3-fold. When the number of nodes increases 4-fold, the reductions in runtime are 3.2- and 1.5-fold for the construction and simplification, respectively. The experiments show that Graph Construction tended to possess superior scalability in MapReduce.

## Discussion and conclusions

With the rapid growth of sequence data, genome assembly remains one of the most challenging computational problems in genomics. String graph-based approaches have the benefits of read coherence [11], less memory requirement, and successful experience in analyzing Sanger sequence data [23]. In this report, we identify several types of structural defects in string graphs resulting from sequencing errors and short repeats. To remedy the structural defects in string graphs, we developed the EA algorithm that utilizes information from the consensus of graphical neighbors. To validate the effectiveness of the EA algorithm, we used simulated data to define four types of edges and a braid index to help evaluate the structural defects in string graphs. The experimental results show that the EA algorithm efficiently minimizes structural defects in string graphs. Thus far, the EA algorithm is not suitable for studies on SNPs, because it only removes the edges. We suggest that correcting the edges with sequence logos will maintain information for SNP analysis; this is the subject of a future study.

To demonstrate the validity of CloudBrush, we used GAGE benchmarks [18] to compare CloudBrush with other state-of-the-art assembly tools. The evaluation results show that CloudBrush is a conservative assembler that nevertheless can generate precise contigs that avoid error propagation in downstream analysis with moderate N50 contig lengths. We also tested the scalability of CloudBrush using three different sizes of hadoop clusters to assemble ~7-Gbp data of the *C. elegans *dataset on a hicloud™ computing service [19]. The study results show that the stage of graph construction is the primary performance bottleneck and its scalability in the MapReduce framework is quite impressive.

In future studies, we will incorporate the scaffolding issue and mate-pair analysis into the MapReduce pipeline. Combining state-of-the-art error correction and our edge analysis is another subject worthy of investigation. We believe that CloudBrush will achieve a better contig N50 with fewer misjoin errors if these former two issues are resolved. Adapting the pipeline toward third generation sequencing technologies is also an important direction of investigation.

## Methods

We previously described a string-graph base assembly algorithm using MapReduce called CloudBrush [15]. The framework of MapReduce can easily be implemented as a modular pipeline, allowing it to be easily extended when improved algorithms have been developed. In this study, we have expanded on CloudBrush by revising its pipeline and adding an EA algorithm. We introduced the principle of the graph processing in MapReduce and the pipeline of CloudBrush. It is noteworthy that the code is written in Java and readers may refer to [15] for further details concerning the implementation of the procedures in the MapReduce framework.

### Distributed graph processing in MapReduce

Genome assembly has been modelled as a graph-theoretic problem. Graph models of particular interests include de Bruijn and string graphs in either directed or bidirected forms. Here we use bidirected string graph to model the genome assembly problem.

In a bidirected string graph, nodes represent reads and edges represent the overlaps between reads. To model the double-stranded nature of DNA, a read can be interpreted in either forward or reverse-complement directions. For each edge that represents an ordered pair of nodes with overlapping reads, four possible types exist, according to the directions of the two reads: forward-forward, reverse-reverse, forward-reverse, and reverse-forward. The type attribute is incorporated into each edge of the bidirected string graph. It is noteworthy that traversing the bidirected string graph should follow a consistent rule, i.e., the directions of in-links and out-links of the same node should be consistent. In other words, the read of a specific node can only be interpreted in a unique direction in one path of traversal.

The MapReduce framework [16,17] use *key-value *pairs as the only data type to distribute the computations. To manipulate a bidirected string graph in MapReduce, we use a *node adjacency list *to represent the graph, which stores *node id *(i.e., the identifier of a node) as the *key*, and *node data structure *as the *value. Node data structure *contains features of the node as well as a list of its outgoing edges and their features. The *node adjacency list *is a compact representation and allows easy traversal along the outgoing links. In MapReduce, a basic unit of computations is usually localized to a node's internal state and its neighbors in the graph. The results of computations on a node are emitted as *values*, each *keyed *with the identification of a neighbor node. Conceptually, we can think of this process as "passing" the results of computation along out-links. In the reducer, the algorithm receives all partial results having the same destination *node id*, and performs the computation. Subsequently, the data structure corresponding to each node is updated and written back to distributed file systems.

### CloudBrush: string graph assembly using MapReduce

**Figure 11:**
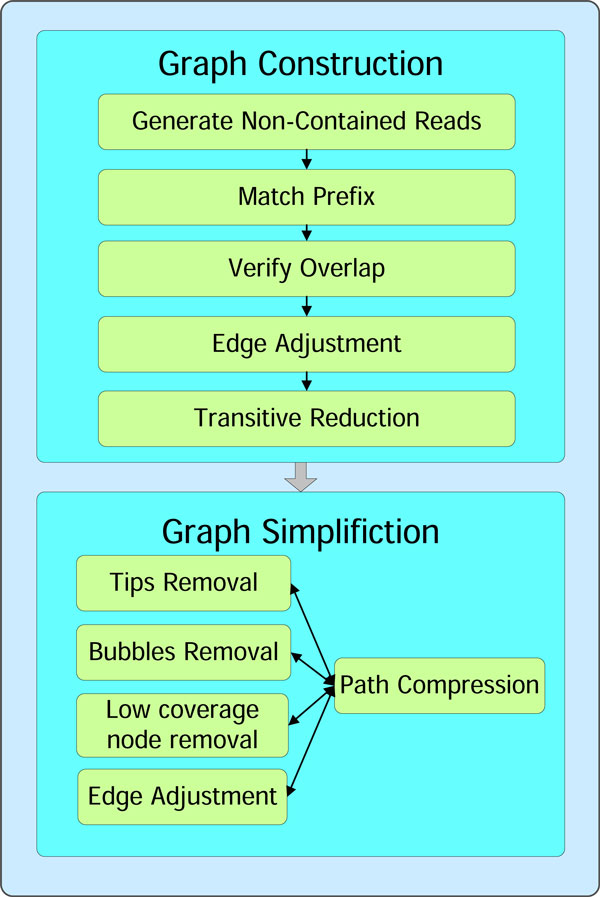
**Workflow of CloudBrush assembler with Edge Adjustment**.

Since Edge Adjustment can effectively and efficiently manage the complex graph structures (see Tables 1 and 2), we remove the path search and SNEA operation modules, which were used to manage braid structures and were the scalability bottleneck in the previous version. Thus, the new pipeline of CloudBrush is summarized as follows: First, we constructed the string graph in four steps: retaining non-redundant reads as vertices, finding overlaps between reads, performing edge adjustment, and removing redundant transitive edges. Second, we simplified the string graph by compressing non-branching paths, removing tips and bubbles using algorithms similar to those used by Contrail [10], and reusing Edge Adjustment as an option to simplify the graph further. Figure 11 displays the workflow of CloudBrush.

### Graph construction in MapReduce

#### 1. Retaining non-redundant reads as vertices

A sequence read may have several redundant copies in the dataset by oversampling in Solexa or SOLiD sequencing. The first step in graph construction is to merge redundant copies of the same read into a single node. We implemented a distributed prefix tree in MapReduce to extend Edena's prefix-tree approach [12].

#### 2. Finding pairwise overlaps between reads

Read-read overlaps are basic clues in connecting reads to contigs; however, finding overlaps between reads is often the most computationally intensive step in string graph-based assemblies. To find all the pairs of read-read overlaps, we adopted a prefix-and-extend strategy to speed up construction of the string graph [15]. The strategy consists of two phases, the prefix phase and the extend phase. In the prefix phase, a pair of reads is reported if the prefix of one of the reads exactly matches a substring of the other read at the given seed length. The pair is then said to have a "brush." In the extend phase, pairs of reads having a brush are further validated starting from the brush. If the exact match extends to one end of the second read, then an edge containing the two nodes of the two reads is created.

#### 3. Edge Adjustment

**Figure 12:**
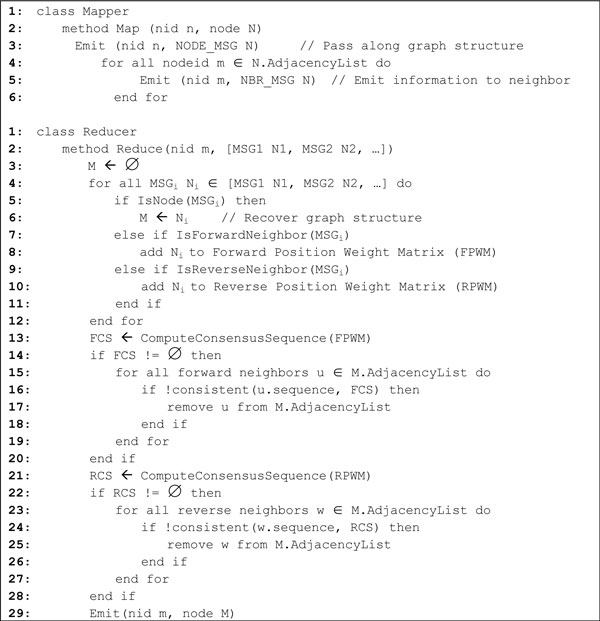
**The pseudo code of the EA algorithm in MapReduce version**.

After finding overlaps as edges, we used the EA algorithm on the graph structure. To perform the EA algorithm in the MapReduce framework, we passed the neighbors' edges for each node r*_i _*such that r*_i _*knows all of the neighboring nodes in the reducer. Once a node possesses all of the neighbors' information, the EA algorithm can easily compute the consensus sequence from the neighbors' content and perform the edge adjustment as described in Results sections. Figure 12 shows the pseudo code of the Edge Adjustment algorithm in MapReduce version. It is noteworthy that, in MapReduce framework, each node computes its own consensus sequence in parallel.

#### 4. Reducing transitive edges

After the EA algorithm, the graph still has superfluous edges due to oversampling in sequencing. Consider two paths of consecutively overlapping nodes r*_a_*→r*_b_*→r*_c _*and r*_a_*→r*_c_*; r*_a_*→r*_c _*is transitive because it spells the same sequence as r*_a_*→r*_b_*→r*_c_*, but ignores the middle node r*_b_*.

To perform the transitive reduction in the MapReduce framework, we passed the neighbors' edges for each node r*_i _*such that r*_i _*knows all the neighboring nodes in the reducer. Different from de Bruijn graphs, the overlap size information is attached to the edge of our bidirected string graph. Therefore, we can sort neighbors by overlap size and remove transitive edges in order.

### Graph simplification in MapReduce

After constructing the string graph, we used several techniques to simplify the graph, including path compression, tip and bubble removal, and low coverage node removal. Path compression is used to merge a chain of nodes, each having one in-link and one out-link along a specific strand direction into a single node. After path compression, tips and bubbles are easily recognized locally. Our MapReduce implementation of path compression, tip and bubble removal, and low coverage node removal are similar to that of Contrail [10], except that we add an additional field of overlap size for the data structure of message passing between nodes tailed for the string graphs. Additionally, we provide an option to reuse the EA algorithm in graph simplification. In this study, we only performed the EA algorithm on nodes whose neighbors were dead ends of the graph; more broadly, the EA algorithm can be performed iteratively until no dead-end neighbors can be removed.

## Competing interests

The authors declare that they have no competing interests.

## Authors' contributions

YJC and CCC were equal contributors in developing the whole idea and writing the manuscript. CLC and JMH were leaders of the team and participated in the design of the study and revising the manuscript. All authors read and approved the final manuscript.
